# Book Reviews

**Published:** 1896-04

**Authors:** 


Manual of Gynecology. By Henry T. Byford, Professor of Gynecology and Clinical Gynecology in the College of Physicians and Surgeons of Chicago; Professor of Clinical Gynecology in the Woman's Medical School of Northwestern University; Professor of Gynecology in the Post-Graduate Medical School of Chicago. Octavo, pp. xii.—488, containing 234 illustrations, many of which are original. Price, $2.50, Philadelphia: P. Blakiston, Son & Co., 1012 Walnut street. 1895.

This book has been written by an author of experience and cannot fail to prove instructive to the student. We are among those who believe that the teaching of gynecology need not commence until the student's final year and then should be largely clinical. In this statement, too, we are presuming upon a four years' course.

Byford has separated the practical from the technical by the use of different kinds of type, a plan which we commend. On page 19 are seven drawings of postures made from original photographs taken by the writer and published in the Transactions of the American Association of Obstetricians and Gynecologists, Volume V., 1892. Credit to the original source, however, is omitted, which we presume is an oversight.

We think it would have been better if Byford had prepared his manual exclusively for students, and thus kept it within the range of elementary gynecology. However, we still regard it as one of the best manuals published and commend it to the favor of students.

---

PHYSICIANS AND SURGEONS OF AMERICA. A Collection of Biographical Sketches of the Regular Medical Profession. Edited and Compiled by IRVING A. WATSON, A. M., M. D., Secretary American Public Health Association, etc. Imperial 8vo, pp. 843. Illustrated. Concord, N. H. Republican Press Association. 1896.

This long-expected volume has at last appeared. It has been announced several times, but of necessity there is unavoidable delay in the issuance of such a book. It will be doubly welcome to the medical profession, because, first, it is one of the most complete of its kind; and second, the editor has excluded sketches of irregular practisers of medicine.

There are several points of excellence to be commended in this volume. In the first place, the biographies are accompanied by excellent half-tone pictures. It is quite important to know how a man looks when reading his own contributions to medical literature, or when writing sketches of his work for publication in medical journals. Again, the biographies are for the most part extremely accurate, being confined to chronological data and are devoid of fulsome praise.

The general plan of the work is exceedingly good and its mechanical execution is without fault or blemish. It is printed on number one book paper, and is bound in a substantial as well as tasteful manner. It is to be regretted, however, that many men whose biographies ought to be here included have declined or failed to furnish them. We can understand the feeling that has prompted many to this course. Most self-respecting physicians dislike to have their photographs and biographies published alongside of even regular physicians whose methods are questionable, to say nothing of irregulars and quacks. A second edition of this volume will, no doubt, contain very many additional names and pictures, for it will soon be discovered that objectionable features have been scrupulously excluded.

This book will prove of vast assistance to medical editors as well as to newspaper men in general, and ought to be placed on the shelves of every public library. The editor is to be congratulated upon the completion of such a great labor in so satisfactory a manner.

PREGNANCY, LABOR AND THE PUERPERAL STATE. By EGBERT H. GRANDIN, M. D., Consulting Surgeon to the New York Maternity Hospital; Consulting Gynecologist to the French Hospital, New York, etc.; and GEORGE W. JARMAN, M. D., Obstetric Surgeon to the New York Maternity Hospital; Gynecologist to the Cancer Hospital, New York, etc. Illustrated, with forty-one (41) original full-page photographic plates from nature. Royal 8vo, pp. viii.—261. Cloth, $2.50 net. Philadelphia: The F. A. Davis Co., Publishers, 1914 and 1916 Cherry street. 1895.

This work differs from any yet issued pertaining to the practice of obstetrics. It is a monograph made up largely of clinical facts which have been demonstrated by the authors—one or both. We believe it will serve to improve the teaching of the obstetric art to have such works as this adopted as a guide.

The book is divided into three parts, as indicated by its title, the first of which, pregnancy, is dealt with in three chapters. Chapter I. considers the diagnosis, differential diagnosis, duration and hygiene of pregnancy; Chapter II., the pathology of pregnancy; Chapter III., the diagnosis of the presentation and the position of the fetus.

The second section treats of labor in four chapters: Chapter I., considering the mechanism of labor; Chapter II., its clinical course; Chapter III., the management of normal and abnormal labor and Chapter IV., the care of the newborn infant.

The third section considers the puerperal state in two chapters, the first treating of the normal puerperium and the second of the pathological puerperium. In the latter chapter we are pleased to note the following: "The term, puerperal fever, may still suffice for the laity. It can have but one meaning for the physician, and this is septicemia, blood-poisoning, wound infection, the absorption of products of decomposition altered through infection into a virulent poison." When authors begin to teach facts in this way we are verily making progress.

The illustrations are among the best we have seen in an obstetrical treatise and are all original.

